# Effectiveness of Resident Physicians as Triage Liaison Providers in an Academic Emergency Department

**DOI:** 10.5811/westjem.2017.1.33243

**Published:** 2017-04-17

**Authors:** Victoria Weston, Sushil K. Jain, Michael Gottlieb, Amer Aldeen, Stephanie Gravenor, Michael J. Schmidt, Sanjeev Malik

**Affiliations:** * Northwestern University Feinberg School of Medicine, Department of Emergency Medicine, Chicago, Illinois; † Rush University Medical Center, Department of Emergency Medicine, Chicago, Illinois; ‡ Center for Emergency Medical Education, US Acute Care Solutions, Chicago, Illinois

## Abstract

**Introduction:**

Emergency department (ED) crowding is associated with detrimental effects on ED quality of care. Triage liaison providers (TLP) have been used to mitigate the effects of crowding. Prior studies have evaluated attending physicians and advanced practice providers as TLPs, with limited data evaluating resident physicians as TLPs. This study compares operational performance outcomes between resident and attending physicians as TLPs.

**Methods:**

This retrospective cohort study compared aggregate operational performance at an urban, academic ED during pre- and post-TLP periods. The primary outcome was defined as cost-effectiveness based upon return on investment (ROI). Secondary outcomes were defined as differences in median ED length of stay (LOS), median door-to-provider (DTP) time, proportion of left without being seen (LWBS), and proportion of “very good” overall patient satisfaction scores.

**Results:**

Annual profit generated for physician-based collections through LWBS capture (after deducting respective salary costs) equated to a gain (ROI: 54%) for resident TLPs and a loss (ROI: −31%) for attending TLPs. Accounting for hospital-based collections made both profitable, with gains for resident TLPs (ROI: 317%) and for attending TLPs (ROI: 86%). Median DTP time for resident TLPs was significantly lower (p<0.0001) than attending or historical control. Proportion of “very good” patient satisfaction scores and LWBS was improved for both resident and attending TLPs over historical control. Overall median LOS was not significantly different.

**Conclusion:**

Resident and attending TLPs improved DTP time, patient satisfaction, and LWBS rates. Both resident and attending TLPs are cost effective, with residents having a more favorable financial profile.

## INTRODUCTION

Emergency department (ED) volumes continue to grow, with a 23% increase over 10 years to 116.8 million visits in 2007.^1^ ED crowding remains a complex and challenging problem for healthcare systems worldwide, with negative impacts upon staff and patient satisfaction, ED wait times, and potentially harmful delays in providing quality patient care.^2^–^4^

Crowding can result from input (patient volume), throughput, and output stressors (ED boarding, inpatient capacity constraints).^5^,^6^ Operationally, throughput is the factor under greatest direct control of the ED, as it represents patient care from ED arrival to disposition. As a result, the majority of interventions directed towards addressing ED crowding have focused on throughput optimization.

Several metrics are commonly used as surrogate measurements for the quality of ED care, including assessments of the timeliness of ED care and patient satisfaction. Timeliness metrics have been defined to include door-to-provider times (DTP) and length of stay (LOS).^7^ As waiting times increase, patients may leave the ED prior to physician evaluation. These patients, categorized as left without being seen (LWBS), can suffer deleterious consequences including death and disability. From an operational standpoint, LWBS also constitutes lost ED revenue and potentially lost hospital revenue if the patient’s condition would warrant further inpatient admission.

Many interventions in EDs across the country have aimed to mitigate the effects of crowding and optimize these metrics. Such efforts have ranged from nurse-initiated triage order sets to ED compartmentalization based on acuity to the installment of advanced practice providers or ED attending physicians in triage to initiate patient workups.^8^ These triage liaison providers (TLPs) work to expedite and initiate the workup of patients, especially those of higher acuity, as well as identify and rapidly assist those of lower acuity who can be cared for without an official ED treatment space. They have the potential to effectively mitigate the consequences of crowding by decreasing DTP, LOS, and LWBS and improving patient satisfaction.

Prior studies have evaluated both attending physicians and advanced practice providers (i.e. nurse practitioners, physician assistants) serving as TLPs with varied results.^10^–^12^,^19^ Many studies have illustrated decreased LOS with TLPs, including a systematic review by Rowe in 2011 including 28 studies that showed a 37-minute decrease in average LOS.^13^–^25^ Others have also shown improved LWBS^12^,^14^–^16^,^18^ and DTP.^13^,^18^ Alternatively, some studies have suggested that having an attending TLP is not feasible due to the increased labor costs, increased staffing needs, and variations in practice between the TLP and end provider.^8^ The few studies on cost effectiveness of TLPs demonstrate a net increase in the cost when using an attending provider.^19^, ^24^

TLPs have been shown to be successful in improving ED metrics including DTP, LWBS, LOS, and patient satisfaction. The majority of these studies have evaluated attending physician or advanced practice providers. To our knowledge, the literature on the impact of resident physician TLPs is limited to a single abstract and a single study indicating decreased LOS without significant change in LWBS.^20^, ^28^ The goal of this study is to compare operational performance metrics, patient satisfaction, and cost-effectiveness outcomes between resident and attending physicians as TLPs.

**Population Health Research Capsule**

What do we already know about this issue?

Prior studies have evaluated attending physicians and advanced practice providers as triage liaison providers (TLP) with mixed results. However, few studies have assessed residents as TLPs.

What was the research question?

What is the difference in operational performance metrics between resident TLPs, attending TLPs, and historical controls?

What was the major finding of the study?

Both attending and resident TLPs improved performance metrics, with residents having a more favorable return on investment.

How does this improve population health?

This article provides information on an alternative staffing model to manage crowding in EDs with emergency medicine residents.

## METHODS

### Study Design

This was a retrospective cohort study that compared predefined aggregate operational performance metrics between resident TLPs, attending TLPs, and a historical control group. This study was approved for exempt status per our institutional review board.

### Setting

This study was conducted at a single urban academic ED associated with a residency program. This ED has approximately 88,000 annual visits and is staffed by 50 residents and 28 attending physicians. The mean admission rate is 20% inpatient admissions and 15% observation admissions. The ED uses electronic medical records (EMR) for all of its encounters and has computerized physician-order entry.

### Selection of Participants

All patients presenting during the hours when a TLP was present were eligible for inclusion. We excluded pediatric patients (defined as age less than 18 years) because they were frequently seen in the nearby dedicated pediatric ED. Senior residents (defined as post-graduate year [PGY] 3 and 4 emergency medicine residents) and attending physicians were eligible for participation as the TLP. Senior resident physicians were staffed as TLPs through voluntary moonlighting. Attending physicians were staffed as TLPs based upon scheduled faculty shifts.

### Interventions

A TLP was present between 11:30 and 19:30 on Monday through Friday from October 2013 through January 2014. Patients were initially evaluated by triage registered nurses (RN) as on non-TLP days. Typical triage flow for RNs included patient evaluation at intake by an initial triage nurse (T1), who would direct immediate placement of critically ill Emergency Severity Index (ESI) 1 or ESI 2 patients as well as immediate placement of ESI 4 and ESI 5 patients into our fast track area, which was open during the day and included the hours a TLP was present. All other patients were then taken to a second intake area and seen by a second triage nurse (T2). On TLP and on non-TLP days, T2 nurses initiated labs off of care-initiation guidelines or in discussion with the TLP physician when present, but did not order any advanced testing. On TLP and non-TLP days, all patients with a chief complaint of chest pain had electrocardiograms (EKGs) performed in triage. These EKGs were then taken to the TLP or an attending physician or senior resident physician on a main ED team for review.

TLP staffing was in a split-flow design. TLPs worked in our second triage intake area with T2 nurses. When staffing permitted, a dedicated RN was assigned to assist the TLP. Due to the high ED volume, TLPs did not see every patient. Typical TLP responsibilities included screening of EKGs, prioritizing placement of ESI 2 patients, care initiation of as many ESI 3 patients as possible, and primary management of select ESI 3 patients. TLPs were not directed to focus on ESI 4 and ESI 5 patients as our ED had a fast-track area open during the same hours. TLPs were able to order all labs, medications, EKGs, and imaging tests as would be ordered during a regular ED evaluation. TLPs wrote brief, 2–3 sentence notes on evaluated patients who were placed in regular ED beds, which were visible by the primary ED team in the EMR. TLPs wrote full ED notes for patients whom they managed primarily. Patients managed primarily by the TLP were evaluated in a private room in triage and then placed back in the waiting room to await test results and imaging. These patients were admitted or discharged from the waiting room.

### Study Protocol

Outcomes for all ED patients on the TLP days (from 10/2013–1/2014) were compared for senior residents and attending physicians. We also compared outcomes to a historical control (defined as pre-TLP data from 10/2011–1/2012). No other major co-interventions were performed during this time period. Outcome data was generated using data from the entire TLP day or non-TLP day, and was not limited to the specific hours that a TLP was present.

### Outcomes

The primary outcome was overall cost effectiveness, defined as the return on investment (ROI). We calculated ROI using the annual revenue based upon the LWBS capture offset by the TLP cost. TLP cost was calculated by multiplying the provider hourly cost by the annual number of hours worked as a TLP. We evaluated revenue capture based on projected physician-based collections and hospital-based collections. Secondary outcomes included differences in median ED LOS (for both admitted and discharged patients), median DTP, percentage of LWBS, and proportion of “very good” overall patient satisfaction scores. For ED LOS, start time for the ED visit was determined by initial arrival and registration into the system. Time of admission and time of discharge were based on the times when the patient physically left the ED, based on the patient’s clinical status change in the EMR to admitted status with an assigned inpatient location, or to discharged status. We tracked median DTP on TLP and non-TLP days by assignment of a physician to the patient in the EMR. On TLP days, TLPs used an icon in the EMR to assign themselves to patients they had seen and provided care for in triage.

### Analysis

Data were extracted and stored through an offsite, secured electronic data warehouse. We described proportions as means with 95% confidence intervals (CI), while the remainder of the data was described with medians with interquartile ranges. We analyzed data with t-tests for data with normal distribution and the Mann-Whitney U test for non-normally distributed data using Stata statistical software (StataCorp Version 13.0; College Station, Texas).

## RESULTS

Over the four-month study period, residents worked 29 days as a TLP and attending physicians worked 48 days as a TLP, for a total of 77 TLP days, compared to 92 historical control days. We analyzed 6,683 visits in the resident group, 10,814 in the attending group, and 19,298 in the historical control group.

**Figure 1:**
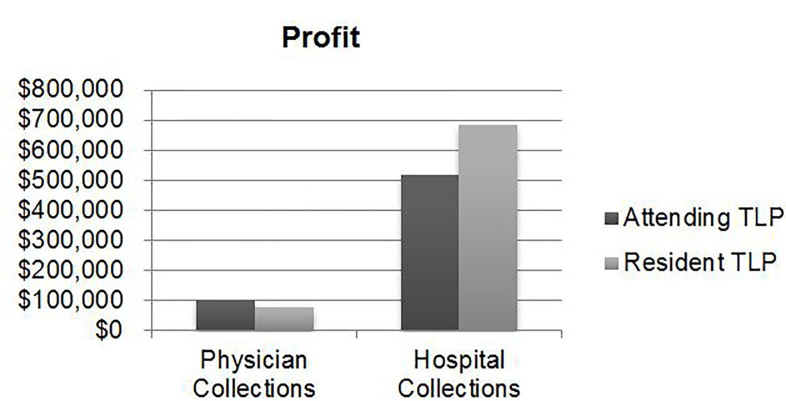
Difference in annual profit generated by attending triage liaison provider (TLP) and resident TLP, through physician collections and hospital collections.

**Figure 2:**
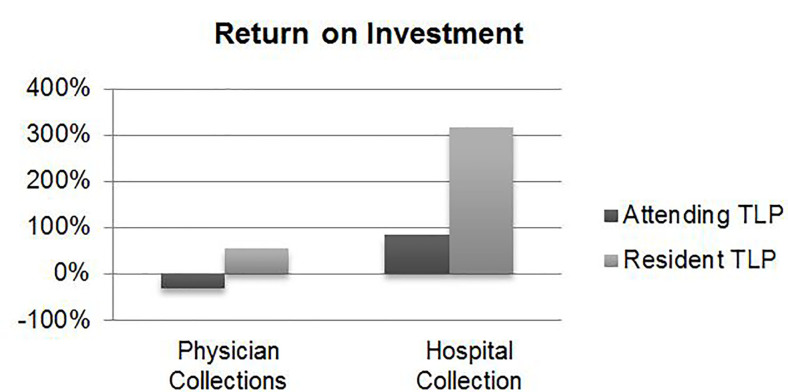
Difference in return on investment for attending triage liaison provider (TLP) and resident TLP, through physician collections and hospital collections.

Annual profit generated for physician-based collections through LWBS capture (after deducting respective salary costs) equated to a gain of $77,997 (ROI: 54%) for resident TLPs and a loss of $104,752 (ROI: −31%) for attending TLPs (Table, Figure 1). Accounting for hospital-based collections made both profitable, generating $684,504 in profit (ROI: 317%) for resident TLPs and $519,467 in profit (ROI: 86%) for attending TLPs (Table, Figure 2).

**Figure 3:**
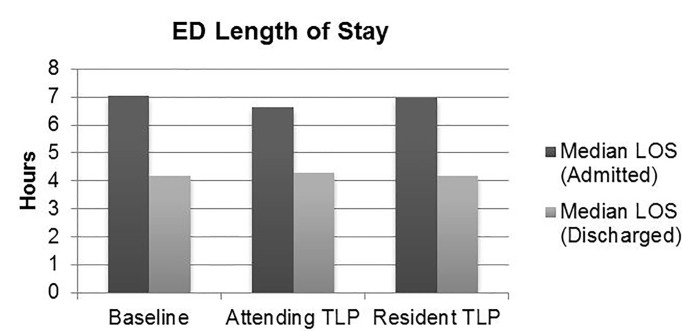
Difference in ED length of stay between attending TLP, resident TLP, and historical control.

Overall median LOS was not significantly different with a TLP compared to historical control (Table, Figure 3). Attending TLPs were associated with a *lower* median LOS for admitted patients compared to resident (6.63 hours vs. 6.97 hours, p=0.004) or historical control (6.63 hours vs. 7.03 hours, p<0.0001). Conversely, attending TLPs were associated with a *higher* median LOS for discharged patients compared to resident TLPs (4.28 hours vs. 4.18 hours, p=0.01) or historical control (4.28 hours vs. 4.17 hours, p=0.0002).

**Figure 4:**
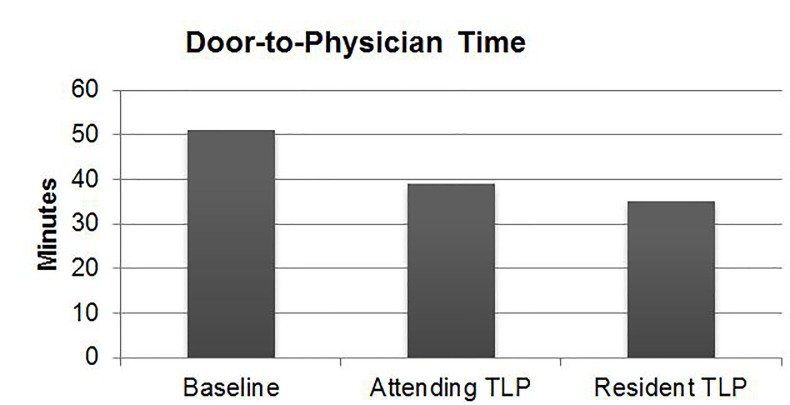
Difference in door-to-physician time between attending triage liaison provider (TLP), resident TLP, and historical control.

Median DTP was significantly lower with a TLP compared to historical control (Table, Figure 4). Median DTP was 35 minutes (interquartile range [IQR] 17–81 minutes) for resident TLPs, significantly lower (p<0.0001) than attending TLPs (39 minutes, IQR 19–87 minutes) or historical control (51 minutes, IQR 21–117 minutes).

**Figure 5:**
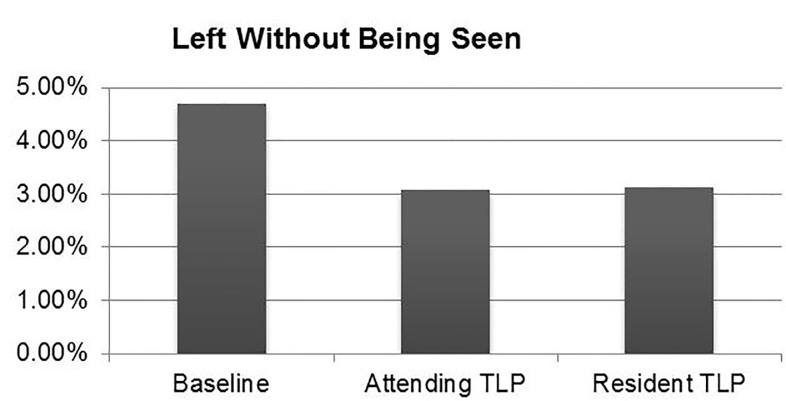
Difference in “left without being seen” percentage between attending TLP, resident TLP, and historical control.

Proportion of LWBS was significantly improved with a TLP compared to historical control (Table, Figure 5). LWBS was 3.12% (95% CI [2.73%–3.55%]) for resident TLPs and 3.08% (95% CI [2.77%–3.41%]) for attending TLPs, both significantly better than historical control (4.71%, 95% CI [4.43%–5.01%]).

**Figure 6:**
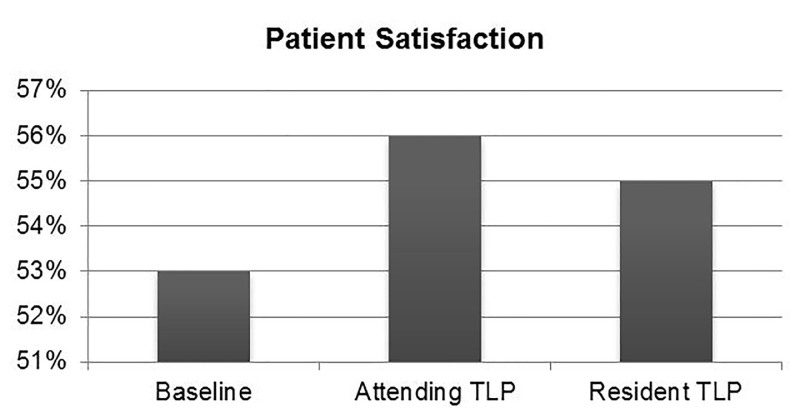
Difference in proportion of “very good” patient satisfaction scores between attending triage liaison provider (TLP), resident TLP, and historical control.

Proportion of “very good” patient satisfaction scores was 55% (95% CI [53%–56%]) for resident TLPs and 56% (95% CI [55%–57%]) for attending TLPs, compared to historical control (53%, 95% CI [52%–54%]). This was not significantly improved with a TLP (Table, Figure 6).

## LIMITATIONS

This was a single center study at an urban academic emergency medicine residency program and thus may not readily generalize to other practice settings. This was also a retrospective design and is subject to all of the potential biases and limitations inherent in this study design. This study was performed over a single four-month period, but there is no reason to suggest that using a different study period would have significantly altered the study results. This study was performed only during high-volume ED times. and it is unclear if similar results would be obtained if a TLP were used during times with lower patient volumes. Our hospital has a separate pediatric ED, so further study would be needed to assess the applicability in pediatric patients. Finally, only senior (PGY 3 and 4) residents were studied as TLPs. Further study is needed before applying this process to more junior residents.

## DISCUSSION

Crowding is a widespread problem that has been increasingly common in many EDs. Studies on crowding have demonstrated negative impacts on patient and provider care, as well as on patient outcomes.^2^–^4^ As a result, EDs have used a variety of techniques to improve throughput and efficiency. One of the more common approaches is to use a TLP, but the majority of studies have assessed only attending physicians and advanced practice providers in this role.^13^–^25^ Our primary outcome was overall cost effectiveness of using a TLP, defined as the ROI.

We are aware of only a few studies assessing the cost effectiveness of using a TLP in triage, none of which assessed resident TLPs. One study was performed in a pediatric ED using an attending pediatric provider as the TLP and suggested an increased cost of $42,883 with this approach.^19^ Another study using attending physicians in an urban county teaching program demonstrated an increased cost of $11.98 per patient.^23^ Alternatively, our study demonstrated that both resident and attending TLPs were cost effective, with resident TLPs being significantly more cost effective than attending physicians and generating a significantly higher ROI. Our study also showed that both resident and attending physician TLPs resulted in improved patient DTP, LWBS, and ROI when compared with historical controls. Additionally, there was no clinically significant difference between attending and resident providers with regard to LOS, DTP, and percentage LWBS.

The effect of TLPs on LOS has been mixed in the literature, though the majority of studies (including a recent systematic review) demonstrate favorable effects on LOS and LWBS.^13^–^25^ To the best of our knowledge, only two prior publications have assessed using residents as a TLP and had conflicting results. An abstract by Porter et al. demonstrated no significant difference in LOS between resident TLPs and standard nursing triage, 28 while a study of 1,346 patients by Svirsky et al. demonstrated decreased LOS without a significant change in LWBS.^20^ Of note, these were both much smaller studies and did not include attending physician TLPs as a comparator. Our study demonstrated only a minimal difference in LOS but a significant difference in LWBS when compared to historical control with minimal difference between attending and resident TLPs. The difference in LOS between our study and priors may be due to a variety of external factors, including number of available ED beds, acutely ill patients preventing the primary provider from seeing or dispositioning the patients, or delays in laboratory or imaging results.

Our study was performed at a large, urban residency-affiliated ED and the results may not apply to other practice settings. Additionally, creating a TLP requires infrastructure, including additional staffing and provider space. However, our study suggests that if a TLP program is already established, allowing resident physicians to serve as TLP may be more cost effective than staffing with attending providers. Given the increasing prevalence of providers serving as TLPs, it may be beneficial for residents to gain experience in this role. Another benefit of using a TLP is the ability to identify abnormal laboratory or imaging findings earlier in the patient presentation, which may theoretically decrease the probability that patients will decompensate during their ED stay. Finally, the increased percentage of “very good” patient satisfaction scores suggests that patients may be more likely to return and refer people to the hospital, which may lead to further unmeasured ROI.

Future studies should include a prospective randomized controlled trial to confirm our findings. Additionally, studies should determine which days and hours are most cost-effective and whether similar outcomes would occur in different practice settings.

## CONCLUSION

In conclusion, both resident and attending physician TLPs improved DTP time, patient satisfaction, and LWBS percentages. Additionally, both resident and attending TLPs are cost effective with residents having a more favorable cost-benefit profile.

## Figures and Tables

**Table:** Comparison of resident TLP, attending TLP, and historical control.

Outcome	Resident TLP	Attending TLP	Historical control
Profit	$77,997	−$104,752	N/A
Return on investment	$684,504	$519,467	N/A
Median length of stay (admitted)	6.97 hours	6.63 hours	7.03 hours
Median length of stay (discharged)	4.18 hours	4.28 hours	4.17 hours
Door-to-physician time	35 minutes	39 minutes	51 minutes
Left without being seen	3.12%	3.08%	4.71%
Patient satisfaction	55%	56%	53%

*TLP*, triage liaison provider.
